# A Retrospective Study on the Adoption of Lipid Management Guidelines in Post-Myocardial Infarction Patients in a Tertiary Care Centre

**DOI:** 10.7759/cureus.41402

**Published:** 2023-07-05

**Authors:** Patricia M Wambua, Zahid Khan, Charles M Kariuki, Elijah N Ogola

**Affiliations:** 1 Internal Medicine, The Nairobi Hospital, Nairobi, KEN; 2 Acute Medicine, Mid and South Essex NHS Foundation Trust, Southend-on-Sea, GBR; 3 Cardiology, Barts Heart Centre, London, GBR; 4 Cardiology and General Medicine, Barking, Havering and Redbridge University Hospitals NHS Trust, London, GBR; 5 Cardiology, Royal Free Hospital, London, GBR; 6 Cardiology, Ubora Heart Service, Nairobi, KEN; 7 Cardiology, The Nairobi Hospital, Nairobi, KEN; 8 Clinical Medicine and Therapeutics, University of Nairobi, Nairobi, KEN

**Keywords:** simvastatin-ezetimibe, retrospective observational study, statin, esc lipid lowering guidelines, stable angina, st-elevation myocardial infarction (stemi), non-st segment elevation myocardial infarction (nstemi), myocardial infarction type 1, myocardial infarction type 2, lipid lowering therapy

## Abstract

Background: Lipid management after acute myocardial infarction (AMI) is one of the important aspects of secondary prevention in the high cardiovascular (CV) risk group, and targeted reduction of low-density lipoprotein cholesterol (LDL-C) remains the primary target for lipid therapy after myocardial infarction (MI).

Study objective: To conduct a retrospective study of the adequacy of lipid management in post-MI patients admitted to a tertiary care centre as compared to the 2019 European Society of Cardiology (ESC) guidelines for the management of dyslipidaemia.

Methodology: The study was a retrospective review of medical records of patients admitted with MI under the Ubora Heart Service, Nairobi Hospital, from January 2020 to June 2022.

Results: The study population included 79 patients, with a mean age of 59.3 (SD ±12), predominantly male (61 patients, 77.2%), and of African descent (60 patients, 75.9%). The majority of the study population presented with an ST-segment elevation myocardial infarction (STEMI) (62%), and the six most prevalent cardiovascular risk factors recorded amongst the patients were: systemic arterial hypertension in 50 (63.3%) patients; dyslipidaemia in 34 (43.0%); type II diabetes mellitus (T2DM) in 25 (31.6); history of smoking in 12 (15.2%); obesity or being overweight in 12 (15.1%); and family history of premature coronary artery disease or sudden cardiac death in four (5.1%) patients. Moreover, 88.6% of the patients had their lipid profile assessment done within 48 hours of admission, with a mean LDL-C level of 3.18 mmol/L (SD ±.18). All the patients recruited in the study were started on high-intensity statins with either 40 mg or 80 mg of atorvastatin or 20 mg or 40 mg of rosuvastatin. Thirty-nine (44%) patients recruited had repeat lipid profiles on follow-up, with a median lipid analysis time of five months (interquartile range (IQR): 2.0-10.0). Of those, only six (17.1%) achieved the LDL-C goal of <1.4 mmo/L while only 16 (45.7%) achieved a greater than 50% reduction from their baseline LDL-C level, with three (8.6%) patients having an increased LDL-C level from baseline. Overall, 14.7% of the patients studied achieved the guideline-recommended LDL-C goal of an LDL-C target of <1.4 mmo/L and a ≥ 50% reduction from baseline LDL-C. After five months of follow-up, 75 (94.9%) patients were on statin monotherapy, with 4 (5.1%) on high-intensity statin and ezetimibe combination therapy.

Conclusion: This retrospective study highlights the need for early sensitisation and the adoption of secondary prevention strategies in acute coronary syndrome (ACS), as recommended by the 2019 ESC guidelines.

## Introduction

Cardiovascular disease (CVD) remains the principal cause of death in adults worldwide, accounting for an estimated 20.5 million deaths annually as of 2021, representing one-third of the global deaths. This is a significant rise from the 12.1 million CVD deaths reported in 1990 [1]. Low- to middle-income countries are contributing disproportionally to these deaths, with four in five CVD deaths occurring in these countries.

Elevated levels of LDL-C play a critical role in the development of atherosclerotic cardiovascular disease (ASCVD) [1]. A strong and direct association exists between LDL-C and the risk of major adverse cardiovascular events (MACE) [2]. Despite the advancement of treatment for acute coronary syndrome (ACS), 20% of ACS survivors experience a subsequent ischaemic cardiovascular (CV) event within 24 months. Furthermore, their five-year mortality rate ranges from 19% to 22% [3]. This could be due to suboptimal control of modifiable risk factors, among other determinants.

Lipid management after myocardial infarction (MI) is one of the critical aspects of secondary prevention in this high cardiovascular (CV) risk group, and targeted reduction of LDL-C remains the primary target for lipid therapy post-MI. The Scandinavian Simvastatin Survival Study (4S), a multicentre, randomised, placebo-controlled trial, confirmed a 30% relative risk reduction in all-cause mortality (p=0.0003) providing evidence of mortality benefit [4]. Surprisingly, further analysis of the 4S study revealed a 20% CV event rate persisted despite the patients being on moderate-intensity statin therapy, a finding seen in other major statin trials, which prompted a focus on optimal statin use [5,6].

Consequently, additional studies evaluated the incremental benefits of high-dose statins in high-risk patients and lower LDL-C targets in reducing CVD risk. The meta-analyses by Cannon compared the reduction of CV outcomes with high-dose statin therapy to standard dosing and found a 16% odds reduction in coronary deaths and myocardial infarction [7]. The 2005 meta-analysis by the Cholesterol Treatment Trialists' (CTT) Collaborators confirmed that statin therapy could safely reduce the five-year incidence of major coronary events, coronary revascularisation, and stroke, irrespective of the initial lipid profile or other presenting characteristics. It demonstrated that the absolute benefit depended on the total reduction in LDL-C and the individual's absolute risk [8].

Consistent data from meta-analysis support the attainment of the most significant LDL-C decrease to prevent future ASCVD, with no lower limit to this effect. The first meta-analysis demonstrating this was by Baigent, who found that a decrease in LDL-C of 1.0 mmol/L (40 mg/dL) leads to a 22% relative risk reduction in the annual major vascular events rate [8]. To further build on this finding, a meta-analysis on >38,000 patients found that patients who attained very low LDL-C levels (<50 mg/dL or 50-70 mg/dL) had a lower significant CVD event risk than those who achieved moderately low levels (75-100 mg/dL), confirming the benefits of even lower LDL-C targets [9].

Lipid-lowering therapy (LLT) has substantiated efficacy in decreasing LDL-C and reducing the risk of ASCVD-related events. Extensive data from randomised controlled trials (RCTs) have confirmed that lowering LDL-C levels is the mainstay of secondary prevention of ASCVD-related events following an acute myocardial infarction (MI) [9,10]. Despite this, attaining guideline-recommended LDL-C goals in the real-world setting remains suboptimal [11,12,13]. With Kenya's rising burden of non-communicable diseases, an effective secondary prevention strategy is crucial to reducing CVS morbidity and mortality after ACS. Studying the level of adherence to guideline recommendations is a critical first step in developing and implementing this strategy.

## Materials and methods

A single-centre retrospective study of patients admitted following a MI under the Ubora Heart Service in the Nairobi Hospital, Nairobi, Kenya, was conducted between January 2020 and June 2022. All patients who were >18 years of age and admitted to the Nairobi Hospital with acute myocardial infarction (AMI) from January 2020 to June 2022 and managed under the Ubora Heart Service were included in the study.

Exclusion criteria included any patient with missing data and those lost to follow-up. The standards against which we studied patients recruited into the study were based on the set standard of practices (based on the 2019 European Society of Cardiology (ESC)/European Atherosclerosis Society (EAC) guidelines) for the management of dyslipidaemia in acute MI (AMI) patients and included lipid profile measurement on all patients admitted with AMI; early initiation of high-intensity statin in all statin-naive AMI patients; the treatment goal was a 50% LDL-C reduction from baseline and an LDL-C goal of <1.4mmol/L; repeat lipid profile measurements to be obtained four to six weeks after acute myocardial syndrome presentation; the addition of ezetimibe and or proprotein convertase subtilisin/kexin type 9 (PCSK9) inhibitors, in that order of preference, to high-intensity statin "for patients who have not reached the lipid reduction target (>50% LDL-C reduction from baseline, or LDL-C <1.4 mmol/L) "or as an alternative lipid-lowering therapy in patients intolerant to statins."

According to health records estimates at the Ubora Heart Service, an average of 40 AMI patients were managed in the year prior to the study. An estimated 100 patients were seen in the 2.5 years of this study. A representative sample was drawn from this finite population, and the sample size was determined as follows:

n= (NZ^2 P(1-P))/(d^2 (N-1)+Z^2 P(1-P) )

Where n' = sample size with finite population correction; N = size of the target population = 100; Z = Z statistic for 95% level of confidence = 1.96; P = estimated proportion of MI patients with target LDL-C level = 50% (no data available); d = margin of error = 5%; n = 80

Records of patients who presented with MI during the study period and were managed under the Ubora Heart Service were collected using consecutive sampling of the intensive care unit (ICU) and high dependency unit (HDU) registers. To ensure all the potential patients were captured during the screening process, we also reviewed the catheterization laboratory register of patients. This allowed us to screen for all the MI patients exhaustively admitted and managed under the Ubora Heart Service during the study period. Once a record of all patients' hospital numbers was made, we recruited only the patients who met the inclusion and exclusion criteria into the study. IBM Statistical Package for Social Sciences (SPSS) software (IBM Corp., Armonk, New York, USA) was used to calculate the paired t-test for the sample of patients; additional data such as BMIs, LDL-C levels, and type of lipid-lowering therapy have been provided in the results section.

## Results

**Figure 1:**
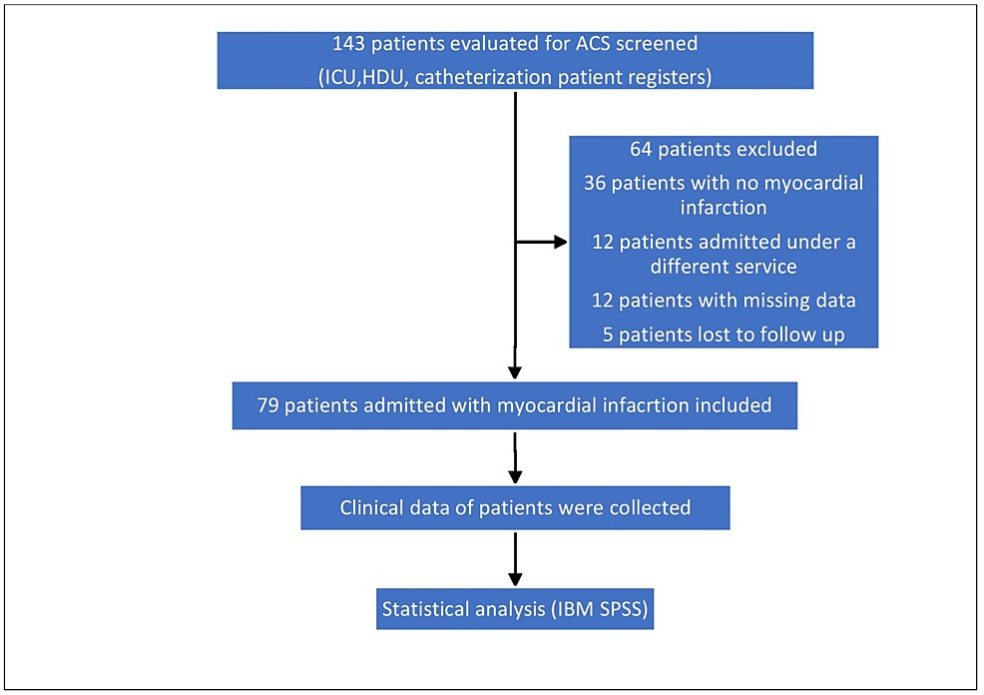
A flowchart of the selection of the patients for the study ICU: intensive care unit; HDU: high dependency unit; IBM SPSS: IBM Statistical Package for Social Sciences (SPSS) statistical software (IBM Corp., Armonk, New York, USA)

Between January 2020 and June 2022, 143 patients were evaluated for ACS and screened for study eligibility, with 64 patients excluded (Figure 1).

This single-centre retrospective cohort included 79 patients, 33-88 years old, hospitalised and managed for an acute coronary event. The mean age of the study population was 59.3 (SD ±12); the youngest patient presenting with ACS was 33 years old, and the oldest was 88 years old. A male predominance of patients managed for ACS during the study period was noted, with 61 (77.2%) of the participants being male. Regarding race, most of the patients managed were African (60, 75.9%), followed by an equal number of Asians and Caucasians (nine, 11.4%). The baseline characteristics of the study population are summarised in Table 1.

**Table 1:** The baseline characteristics of the study population

Baseline demographic variables	Frequency (%)
Gender	
Female	18 (22.8)
Male	61 (77.2)
Age, in years	
Mean (SD)	59.3 (12.0)
Minimum-maximum	33-88
Ethnicity	
African	60 (75.9)
Asian	9 (11.4)
Caucasian	9 (11.4)
Unknown	1 (1.3)

**Figure 2:**
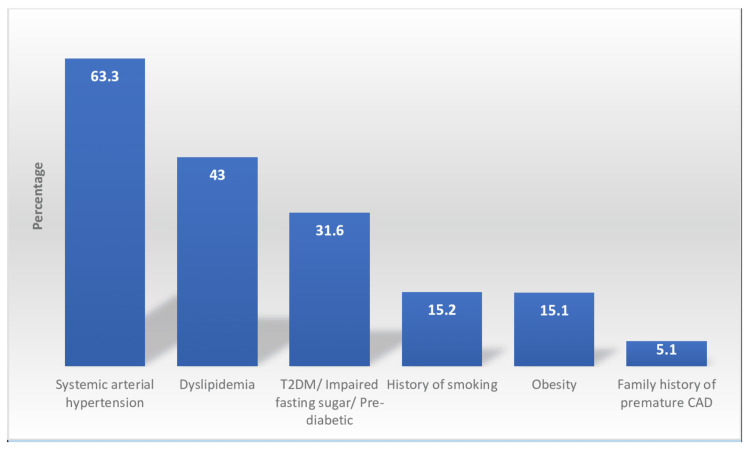
Cardiovascular risk factors in the study population T2DM: type 2 diabetes mellitus, CAD: coronary artery disease

Among the patients managed for ACS, the majority, 49 (62%), presented with an ST-elevation MI (STEMI), while 30 (38%) presented with a non-ST-elevation myocardial infarction (NSTEMI). The six most prevalent cardiovascular risk factors recorded amongst the patients recruited in the study were: 50 (63.3%) patients with systemic arterial hypertension, 34 (43%) with dyslipidaemia, 25 (31.6%) with type II diabetes mellitus, 12 (15.2%) with a history of smoking, 12 (15.1%) who were obese or overweight, and four patients (5.1%) with a family history of premature coronary artery disease or sudden cardiac death. The risk factor profile of the study population is summarised in Figure 1. The three most common comorbidities noted in the study group were hyperthyroidism, connective tissue disease, and human immunodeficiency virus (HIV) infection (Figure 2).

**Figure 3:**
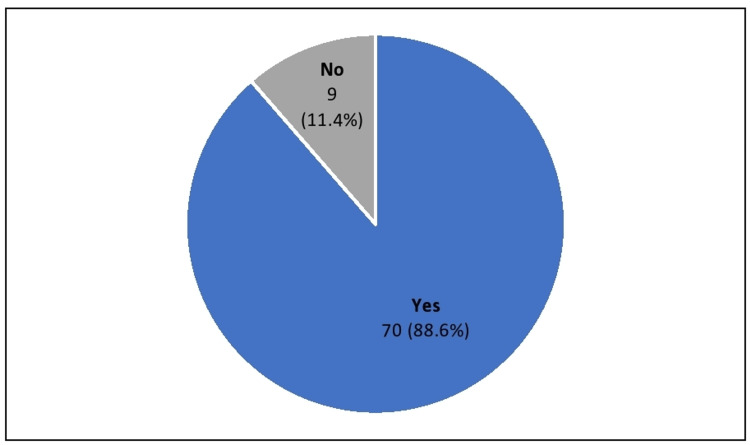
The percentage of the study population with baseline lipid profiles done within 48 hours of presentation with acute coronary syndrome

Of the patients recruited in the study, 88.6% had their lipid profile assessment done within 48 hours of presentation with acute coronary syndrome. We assessed the duration of follow-up, when repeat testing was done, and found a median follow-up time of five months (interquartile range (IQR) of 2-10) post-discharge, with the earliest repeat testing done at three weeks and the most extended follow-up at 29 months (Figure 3).

**Figure 4:**
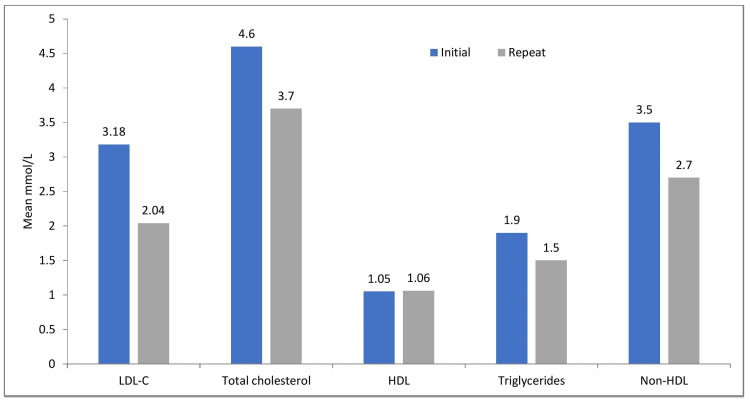
Comparison of baseline versus repeated lipid profile markers

All of the patients admitted with acute MI and recruited into the study were started on high-intensity statins with either 40 mg or 80 mg of atorvastatin or 20 mg or 40 mg of rosuvastatin, with atorvastatin being the most commonly used statin prescribed on admission and maintained at five months post-discharge (75, 94.9%). On review of the patient follow-up, a majority, 44 (55%), of the patients had no repeat lipid profile available on the web-based laboratory reporting system. This precluded the calculation of the LDL-C percentage drop from the baseline variable levels in these patients. Therefore, LDL-C goal assessment was only done on patients with repeat lipid profiles (n=35). The mean LDL-C level at baseline and on repeat testing revealed a baseline LDL-C level of 3.18 mmol/L (SD±1.18) and a follow-up repeat LDL-C level of 2.04 (±0.84). In addition to LDL-C, an analysis of the other lipid markers (total cholesterol (TC), triglycerides (TG), high-density lipoprotein cholesterol (HDL-C), and non-high-density lipoprotein cholesterol (non-HDL)) was done. Figure 4 summarises the change from baseline for the various lipid profile markers.

**Figure 5:**
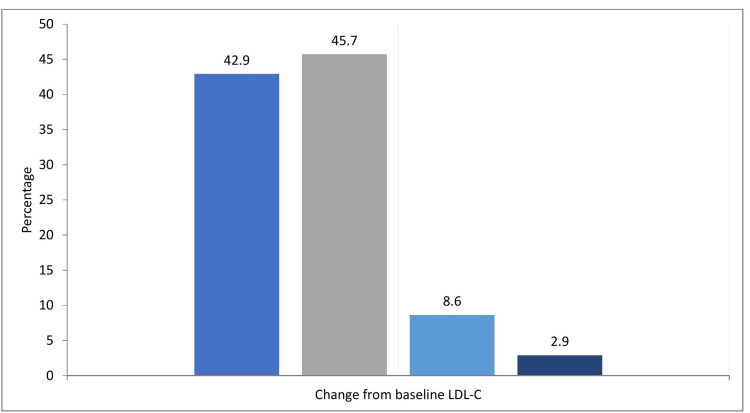
The bar chart shows the percentage change of LDL-C from the baseline

Of those with repeat lipid testing (n=35), 16 (45.7%) of the patients achieved a greater than 50% reduction from their baseline LDL-C level; 15 (42.9%) had a less than 50% reduction; and three (8.6%) of the patients had an increased LDL-C level from baseline, with one patient having no baseline LDL-C evaluated (Figure 5).

When assessing the proportion of patients who attained an LDL-C level below 1.4 mmol/L, only 17.1% achieved this goal, with the majority, 82.9%, having higher LDL-C levels.

**Figure 6:**
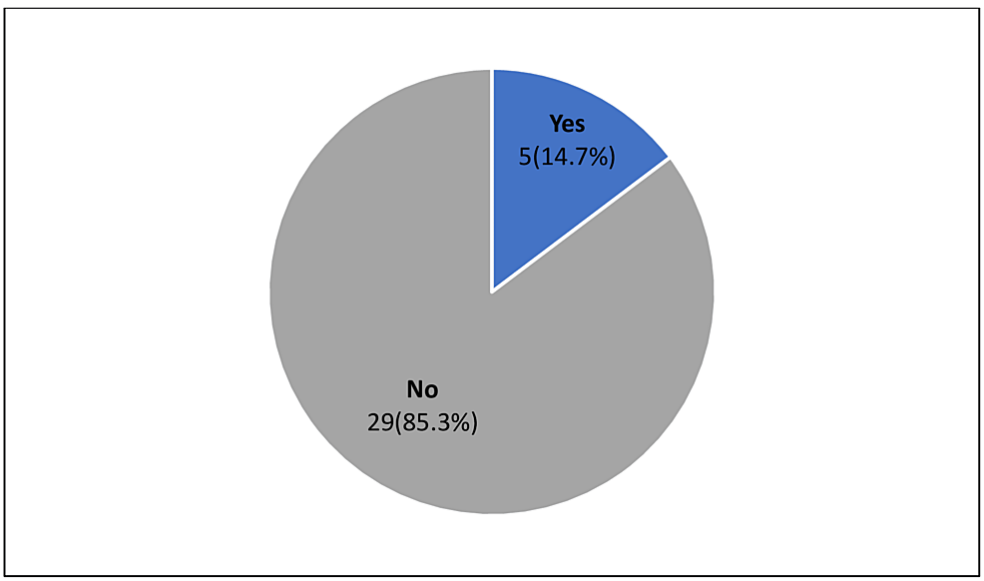
The percentage of patients who achieved the 2019 ESC/EAS LDL-C goal ESC: European Society of Cardiology; EAS: European Atherosclerosis Society

Overall, only five (14.7%) of the patients managed with AMI in our study population achieved the guideline-recommended LDL-C goal of <1.4 mmo/L and a ≥50% reduction from their baseline LDL-C level (Figure 6).

The paired t-test value for the group was 8.76 with degrees of freedom equal to 31 at a significance level α = 0.05. The calculated t-value of 8.76 exceeds the critical value of 2.04, which shows that the means are significantly different.

Lipid-lowering therapy five months following discharge

**Figure 7:**
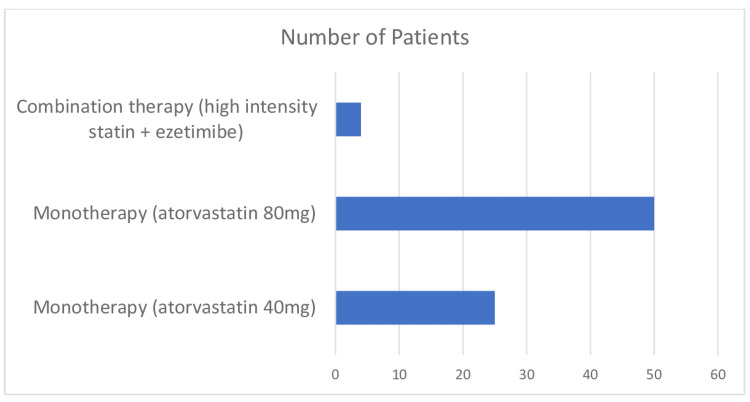
Number of patients and type of lipid-lowering therapy used

On review of the pattern of lipid-lowering therapy use amongst all the patients studied, we found that the majority of the patients, 75 (94.9%), were on statin monotherapy, with only four (5.1%) on a high-intensity statin and ezetimibe combination therapy at five months post-discharge. Moreover, of the 29 patients who did not achieve the LDL-C target, only two were on the ezetimibe-statin combination. Twenty-seven (93.1%) patients remained on monotherapy with a high-intensity statin (Figure 7).

## Discussion

Studies in clinical practice provide an objective assessment of the extent to which the standards set in guidelines on CVD prevention are implemented in everyday practice. Our study is among the first to conduct a retrospective study on dyslipidaemia management in post-myocardial infarction patients in Kenya. The main findings of this retrospective study were commendable: uptake on the use of high-intensity statins in AMI patients; rapid lipid testing done within 48 hours of presentation; no standardised follow-up periods for lipid testing, consequently leading to delays in the post-discharge lipid testing with a median follow-up of five months; the 2019 ESC/EAS guideline-recommended LDL-C goal of less than 1.4 mmol/L and a 50% reduction from baselines was achieved in less than 15% of the study population. There was underutilization of non-statin lipid-lowering therapy (LLT), such as ezetimibe, as an add-on therapy, particularly in those who did not achieve the target. Altogether, the overall finding of the study found non-adherence to guideline recommendations on various strategies to enhance secondary prevention through lipid management in ACS.

Consistent data from meta-analysis support the achievement of the most significant reduction of LDL-C to prevent ASCVD, with no lower LDL-C limit to this benefit detected. Regrettably, the failure to achieve the guideline-recommended LDL-C goal remains a matter of great concern in the real world [9,10]. Our retrospective study revealed that a minority of the patients studied achieved the guideline-recommended LDL-C target of a 50% LDL-C reduction from baseline and an LDL-C goal of <1.4 mmol/L. Our findings are consistent with existing studies, many of which have demonstrated the suboptimal control of dyslipidaemia as a modifiable risk factor in a high-risk patient population. A real-world registry conducted in non-Western countries examined lipid management in ACS and found that among those patients with an LDL-C at the time of follow-up, more than half (52.9%) did not achieve the target LDL-C level [14]. Findings from the EUROASPIRE V survey revealed an overall unsatisfactory control of lipids in a large proportion of patients [15]. Likewise, a sub-analysis from the EYESHOT, a post-MI registry, evaluated the LDL-C target attainment of > 900 post-MI patients from an Italian cohort. It was reported that a large proportion, >52.5%, had suboptimally controlled LDL-C levels (>1.8 mmo/L) [12]. Comparably, a study investigating the LDL-C goal attainment from a nationwide U.S. registry reported that one in three patients was unsuccessful in attaining the 2011 guidelines goals (LDL-C <1.8 mmo/L, or a ≥50% reduction from baseline). The ratio went up to two in three when they applied the 2016 set criteria (LDL < 1.8 mmol/L and a ≥50% reduction from baseline LDL-C levels), demonstrating suboptimal LDL-C attainments among post-MI patients. When comparing the proportion of patients who achieved the 2016-set LDL-C target before and after the introduction of the 2016 guidelines, they noted an increase in the proportion of patients who achieved the target. They attributed this to the rise in treatment intensity with increased use of ezetimibe. This drug had just been included in the 2016 guidelines following the publication of the IMPROVE-IT trial [16]. The constant observation from these studies reveals that substantial efforts should be undertaken to improve LDL-C goal attainment in patients with coronary artery disease (CAD).

To reduce the risk of recurrent CVD events in a high-risk population, physicians managing post-MI patients need to monitor lipid profiles routinely to act upon the LDL-C levels and optimise LLT if required. The importance of this is highlighted in the recommended follow-up timelines given by the 2019 ESC/EAS guidelines. They recommend that testing be done within 48 hours of presentation, four to six weeks after either the initiation or optimisation of LLT, with subsequent three- to six-month follow-up to ensure adherence. Early lipid assessment within 48 hours allows for accurate assessment of baseline LDL-C levels, as they tend to decrease during the first days of ACS. In our study populations, early lipid assessment was done in the majority (48%) of the patients. We, however, found irregular timelines and delays in lipid profile assessment on follow-up visits. Similar to our findings, the real-world registry conducted in non-Western countries found that more than one-third of the patients did not have a repeat LDL-C follow-up measurement [17].

Evidence demonstrating the utility of high-intensity statins in high-risk individuals is irrefutable and well-documented based on the two meta-analyses of high-intensity statin trials [12,13]. An encouraging finding from this retrospective study was that all the patients who presented with AMI recruited into the study were started on high-intensity statins within 24 hours of presentation, as guidelines recommend. This is commendable, given that a study by Unni conducted in a developed country showed that most of the high-risk patients studied were on moderate-intensity statins, with only a minority on high-intensity statins [16]. Despite this high use of statin therapy among our study population, a minority achieved the LDL-C target. There are likely several reasons that could explain this, including patient non-adherence to a prescribed altered lifestyle and LLT. Interindividual variability in statin therapy could also be a possible cause for our study's findings. Several studies over the years have demonstrated this finding, suggesting other factors like genetics, lifestyle, and other coexisting conditions could play a role in treating dyslipidaemia [18,19].

The 2019 guidelines recommend using non-statin LLT to optimise LDL-C levels in patients on maximum-tolerated statins who do not achieve the LDL-C goal. The results of IMPROVE-IT helped establish the use of ezetimibe in combination with statins as a recommended treatment for high-risk patients with hypercholesterolemia [20]. Even though there was a high prevalence of high-intensity statin use on admission in our study population, a review of follow-up prescriptions at five months post-discharge found that there was suboptimal use of ezetimibe in patients with suboptimal LDL-C levels. With 85.3% of the patients not achieving the LDL-C target, a gap in lipid management is evident. A big caveat to consider is that the period of study was largely during the COVID-19 pandemic when the follow-up of non-communicable diseases suffered globally. However, physician inertia could also have contributed to low LDL-C target attainment. None of the patients recruited for the study were on PCSK9 inhibitors. The utilisation of this practical lipid-lowering drug class remains limited due to its high cost and the need for importation from developed countries.

The results of this contemporary study may be considered "real-life data", mirroring contemporary practice among physicians and cardiologists. Alarmingly, the setting of this study was an elite tertiary centre, and the situation is likely to be worse in other settings. They highlight the challenge of improving current practices in managing LDL-C levels as a secondary prevention strategy per guideline recommendations. They also demonstrated the need for implementation science to close the gap between what we know is best and what we practice.

Limitations and recommendations

The small sample size limited our study. This prevents the findings from being extrapolated and reduces the generalisability of the results. In addition, the retrospective review of patient records introduces selection bias. This is because we could only assess the LDL-C percentage drop on the available repeat lipid profile at various follow-up times. A number of international patients managed were repatriated to their respective countries with no access to their follow-up lipid testing. Some local patients might also have done their lipid testing at other laboratories that are not captured in their clinic files. Furthermore, our study did not explore the possible reasons for the low attainment of LDL-C targets.

Larger studies are required to assess lipid management in MI patients by recruiting patients from ACS registries. Data from these real-world registries can potentially improve secondary prevention strategies by raising the awareness of physicians and the entire community about the need for efforts in the secondary prevention of ACS patients. As seen in several studies, ezetimibe, when combined with statins, results in effective LDL-C level reduction. Our study found low use of this combination therapy among this high-CV-risk population that would benefit from a reduction in their total CV risk as a result of the LDL-C reduction achieved with combination therapy. Therefore, we recommend using the highest tolerable statin dose with this reasonably safe and accessible lipid-lowering drug in those patients who are not on target.

Targeted lipid management is a multistep process dependent on regular lipid testing to assess if LDL-C goals are being met. This can only be achieved with proper dissemination, sensitisation, and protocolization of guideline-recommended LDL-C targets, lipid monitoring, and LLT optimisation after hospitalisation for an MI with regular follow-up clinical audits.

## Conclusions

In this retrospective study of contemporary cardiology practice, there was good uptake of high-intensity statins among patients with AMI. However, there were delays in post-discharge lipid testing and suboptimal attainment of the LDL-C goals. There were delays in post-discharge lipid testing, sub-optimal attainment of the LDL-C goal, and underutilization of non-statin lipid-lowering therapy amongst those who needed optimisation, highlighting some gaps in the secondary prevention of patients with previous MI as recommended by the 2019 ESC guidelines. This study could be a useful starting point for further research into this area among patients who need optimisation, highlighting some gaps in the secondary prevention of patients with a previous MI as recommended by the 2019 ESC guidelines.
